# Persistent pain alters AMPA receptor subunit levels in the nucleus accumbens

**DOI:** 10.1186/s13041-015-0140-z

**Published:** 2015-08-12

**Authors:** Chen Su, James D’amour, Michelle Lee, Hau-Yeuh Lin, Toby Manders, Duo Xu, Sarah E. Eberle, Yossef Goffer, Anthony H. Zou, Maisha Rahman, Edward Ziff, Robert C. Froemke, Dong Huang, Jing Wang

**Affiliations:** Department of Anesthesiology, The Third Xiangya Hospital, Central South University, Changsha, Hunan China; Departments of Otolaryngology and Physiology and Neuroscience, The Helen and Martin Kimmel Center for Biology and Medicine at the Skirball Institute for Biomolecular Medicine, New York University School of Medicine, New York, NY USA; Department of Anesthesiology, New York University School of Medicine, New York, NY USA; Columbia College, New York, NY USA; Department of Biochemistry, New York University School of Medicine, New York, NY USA; Departments of Anesthesiology, Neuroscience and Physiology, New York University School of Medicine, New York, NY USA

## Abstract

**Background:**

A variety of pain conditions have been found to be associated with depressed mood in clinical studies. Depression-like behaviors have also been described in animal models of persistent or chronic pain. In rodent chronic neuropathic pain models, elevated levels of GluA1 subunits of α-amino-3-hydroxy-5-methyl-4-isoxazolepropionic acid (AMPA) receptors in the nucleus accumbens (NAc) have been found to inhibit depressive symptoms. However, the effect of reversible post-surgical pain or inflammatory pain on affective behaviors such as depression has not been well characterized in animal models. Neither is it known what time frame is required to elicit AMPA receptor subunit changes in the NAc in various pain conditions.

**Results:**

In this study, we compared behavioral and biochemical changes in three pain models: the paw incision (PI) model for post-incisional pain, the Complete Freund’s Adjuvant (CFA) model for persistent but reversible inflammatory pain, and the spared nerve injury (SNI) model for chronic postoperative neuropathic pain. In all three models, rats developed depressive symptoms that were concurrent with the presentation of sensory allodynia. GluA1 levels at the synapses of the NAc, however, differed in these three models. The level of GluA1 subunits of AMPA-type receptors at NAc synapses was not altered in the PI model. GluA1 levels were elevated in the CFA model after a period (7 d) of persistent pain, leading to the formation of GluA2-lacking AMPA receptors. As pain symptoms began to resolve, however, GluA1 levels returned to baseline. Meanwhile, in the SNI model, in which pain persisted beyond 14 days, GluA1 levels began to rise after pain became persistent and remained elevated. In addition, we found that blocking GluA2-lacking AMPA receptors in the NAc further decreased the depressive symptoms only in persistent pain models.

**Conclusion:**

Our study shows that while both short-term and persistent pain can trigger depression-like behaviors, GluA1 upregulation in the NAc likely represents a unique adaptive response to minimize depressive symptoms in persistent pain states.

## Background

Depression affects up to 100 % of chronic pain patients, and numerous studies suggest that depressed mood accompanies postoperative pain as well [1–7]. Depression leads to additional emotional and cognitive deficits, further impairing recovery and rehabilitation from surgery or injury [8]. While there is evidence that depression alters the threshold of pain, only a limited number of studies have examined whether depression is an integral affective component of the pain experience [9–14]. Pain and depression often co-exist in patients, making it difficult to distinguish a causal relationship. Animal studies provide an opportunity to detect the causal relationship between pain and depressive symptoms and to dissect the molecular mechanisms that regulate this relationship. In rodents, depression-like behaviors can be assessed using the classic sucrose preference test (SPT) [15], and a number of studies have begun to demonstrate that chronic pain in rats leads to depression-like behaviors [16–20].

Imaging studies have shown that pain activates the nucleus accumbens (NAc) [21–23], a brain region well-known to mediate reward-driven behaviors [24, 25]. At the circuit level, the NAc forms reciprocal projections with the amygdala, prefrontal cortex (PFC) and hippocampus – critical regions for pain and depression [18, 20, 26–29]. Recently, neurotrophic, metabolic, transcriptional and epigenetic signaling mechanisms in the NAc have been discovered to regulate depressive behaviors in animal studies [30–34]. Given its established role in depression and its circuit connection to other affective pain centers, the NAc may be expected to contribute to the regulation of pain-induced depression. Molecular changes within the NAc, however, are still not well-defined in pain states.

A previous study shows that the trafficking of GluA1 subunits of the α-amino-3-hydroxy-5-methyl-4-isoxazolepropionic acid (AMPA) receptor in the NAc represents a critical synaptic mechanism in the regulation of chronic neuropathic pain [17]. AMPA receptors, which are the main excitatory postsynaptic receptors for glutamate, are comprised of four distinct subunits, GluA1-4, and subunit composition is crucial to receptor function. Changes in GluA1 subunits at the synapses, specifically, have been shown to strongly regulate depression-like behaviors in a number of animal models [32, 35–39]. GluA1 and 2 subunits are the predominant subunit types in the NAc. Chronic neuropathic pain has been shown to increase GluA1 levels at the NAc synapses, without a concurrent change in GluA2 levels. This selective increase in GluA1 levels leads to the formation of GluA2-lacking AMPA receptors [40–42]. Transmission through these GluA2-lacking AMPA receptors in the NAc, in turn, reduces the depressive symptoms of pain [17]. Thus, AMPA receptor trafficking and signaling dynamics represent a powerful endogenous mechanism to help maintain normal hedonic response in the context of neuropathic pain, likely as an adaptive response to pain. It is not clear, however, whether GluA1 upregulation in the NAc represents a unique synaptic adaptation to neuropathic pain. Neither is it known if this form of synaptic plasticity is also found with transient or reversible pain conditions. The answer to these questions can enhance our understanding of chronic pain at the synaptic and circuit level in the brain.

In the current study, we compare sensory and affective symptoms of three different pain models in rats. We use the paw incision (PI) model to mimic short-term post-incisional pain [43]. We use the Complete Freund’s Adjuvant (CFA) model to mimic persistent but reversible inflammatory pain, and the spared nerve injury (SNI) model to mimic chronic or long-lasting neuropathic pain. We find that in all three pain models, depression-like behaviors develop concurrently with sensory allodynia, suggesting that depressive and sensory symptoms of pain co-exist. GluA1 upregulation, however, is only found with persistent or chronic pain, as it is not found in the PI model. Furthermore, as pain begins to resolve in the CFA model, GluA1 level also returns to baseline. In contrast, in the SNI model, where pain persists for at least 14 days, GluA1 level remains elevated. In addition, a blockade of GluA2-lacking AMPA receptors in the NAc has no effect on the pain behaviors in the PI model, but it further decreases the depressive symptoms in the CFA and SNI models. These results suggest that GluA1 elevation represents a unique dynamic synaptic adaptation to the persistence of pain. This dynamic response provides insight into the role the brain’s reward system plays in chronic pain, and it can potentially serve as a useful molecular marker for the chronicity of pain.

## Results

### Post-incisional pain causes depression-like behaviors in rats

**Fig. 1:**
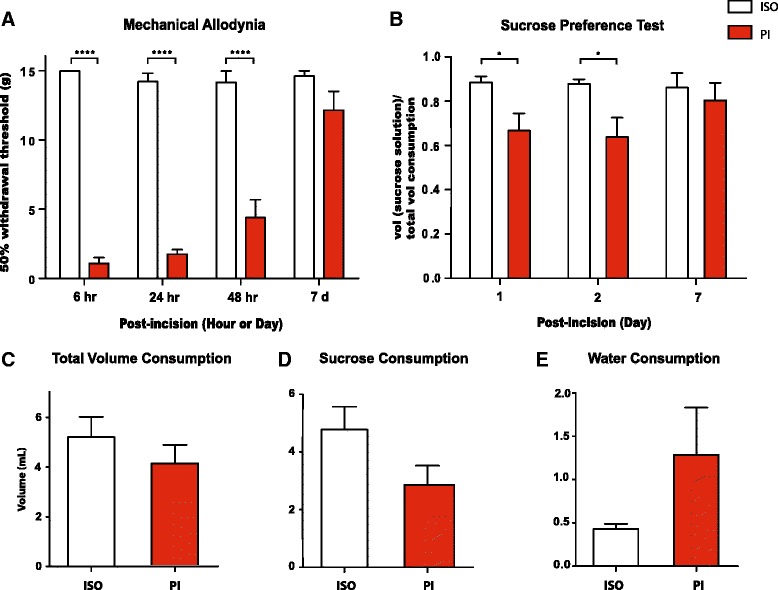
Paw incision (PI) leads to short-term pain and depression-like behaviors. **a** PI-treated rats developed mechanical allodynia up to two days after surgery, compared with control rats that underwent isofluorane treatment without surgery. Two-way ANOVA with Bonferroni post-test, *n* = 6, ****p < 0.0001. On day 7 after PI, there was no statistical difference in the withdrawal threshold between the PI and control groups. **b** PI decreased sucrose preference on the SPT on day 1 and 2 after the procedure, Two-way ANOVA with Bonferroni post-test, *n* = 10, *P < 0.05. **c** PI caused no changes in total fluid consumption on the SPT 1 day after surgery. Student’s *t*-test, *n* = 10, *p* > 0.05. **d** PI caused a decrease trend of sucrose consumption on the SPT 1 day after surgery. Student’s *t*-test, *n* = 10, *p* > 0.05. **e** PI caused an increased trend of water consumption on the SPT 1 day after surgery. Student’s *t*-test, *n* = 10, *p* > 0.05. Error bars show mean and SEM

We used the Brennan (PI) model to mimic post-incisional pain in rats [43, 44]. Here, we incised the right hind paw and measured mechanical allodynia in this paw over the next seven days. As reported previously, mechanical allodynia, which indicated the sensory component of pain, developed quickly after paw incision (<6 h) (Fig. 1a, *p* < 0.0001) [43, 44]. Mechanical allodynia lasted two days, and as described previously, these sensory pain symptoms resolved by the 7^th^ day post-incision (Fig. 1a). In contrast, control rats that had only undergone isofluorane anesthesia treatment without surgical incision did not display any mechanical allodynia.

Next, we measured sucrose preference in rats that experienced PI vs. control rats. Sucrose preference test (SPT) is a classic test for depression-like behaviors in rodents [15]. Specifically, a decrease in rats’ preference for sucrose, a natural reward, indicates anhedonia, a hallmark feature for depression that is pathognomonic in human patients. Compared to control rats, rats in the PI group demonstrated a significant decrease in sucrose preference on day 1 and 2 after incision (Fig. 1b, *p* < 0.05). This level of decrease in sucrose preference is very similar to what has been previously reported for chronic neuropathic pain models [17]. This decrease in sucrose preference was not due to changes in the PI-treated rats’ ability to drink, as there was no statistically significant difference between the control and PI groups in the volume of total fluid consumption (Fig. 1c, *p* > 0.05). The PI group consumed a smaller volume of sucrose solution than control, and these rats drank more water during the test (Fig. 1d, e, *p* > 0.05). These changes were not statistically significant. Together, however, decreased sucrose consumption and increased water consumption result in a statistically significant decrease in sucrose preference in the PI group, suggesting the symptom of pain-induced depression [15].

From a kinetic standpoint, sucrose preference was depressed for two days and returned to control levels 7 days after PI (Fig. 1b). This time-course of decline and return to normal sucrose preference is remarkably consistent with the development and subsequent resolution of mechanical allodynia (Fig. 1a). These results suggest that post-incisional pain can trigger short-term reversible depression-like behaviors.

### Persistent inflammatory pain causes depression-like behaviors in rats

**Fig. 2:**
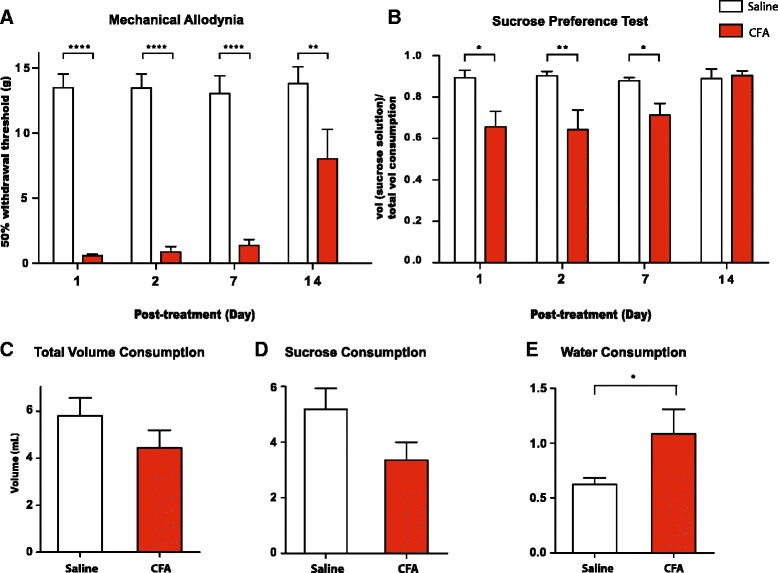
Complete Freund’s Adjuvant (CFA) causes persistent inflammatory pain and depression-like behaviors. **a** Rats developed mechanical allodynia after CFA injection, compared with control (saline) injection. Two-way ANOVA with Bonferroni post-test, *n* = 6–9, **p < 0.01, ****p < 0.0001. **b** Rats developed decreased sucrose preference on the SPT on day 1, 2 and 7 after CFA injection. Two-way ANOVA with Bonferroni post-test, *n* = 6–11, *p < 0.05, **p < 0.01. **c** CFA caused no changes in total fluid consumption on the SPT 7 days after injection. Student’s *t*-test, *n* = 10–11, *p* > 0.05. **d** CFA caused a decreased trend of sucrose consumption on the SPT 7 days after injection. Student’s *t*-test, *n* = 10–11, *p* > 0.05. **e** CFA caused increased water consumption on the SPT 7 days after injection. Student’s *t*-test, *n* = 10-11, *p < 0.05. Error bars show mean and SEM

We used the CFA model to study the time course of presentation of depressive symptoms in an inflammatory pain model. We injected 100 μl CFA into the rat’s right hind paw, and this resulted in localized inflammation and pain. Rats developed mechanical allodynia post-CFA injection, whereas control rats receiving saline injection showed no sensory allodynic changes (Fig. 2a, *p* < 0.01). Furthermore, at the dose of CFA in this study, inflammation and associated sensory nociceptive symptoms began to resolve 14 days after a single injection, as indicated by a return of mechanical sensitivity towards normal control threshold (Fig. 2a).

We assessed depressive symptoms of inflammatory pain using the SPT. Compared to control rats, the CFA group demonstrated a significantly decreased sucrose preference on day 1, 2 and 7 after CFA injections (Fig. 2b, *p* < 0.05). Again, total fluid consumption was not different in these two groups of rats (Fig. 2c, *p* > 0.05), suggesting that the difference in sucrose preference was not due to altered ability of CFA-treated rats to consume fluid. Instead, this difference was due to the fact that the CFA group consumed less volume of sucrose solution than control (Fig. 2d, *p* > 0.05), and they drank statistically more water during the test (Fig. 2e, *p* < 0.05).

14 days after CFA injection, sensory pain symptoms began to resolve, as indicated by a significant increase in mechanical threshold (Fig. 2a). On the SPT, meanwhile, the difference in sucrose preference between these two groups completely disappeared 14 days after CFA administration (Fig. 2b, *p* > 0.05). Qualitatively, these results indicate that both sensory and depressive symptoms of pain begin to resolve after 14 days.

### Persistent neuropathic pain causes depression-like behaviors in rats

**Fig. 3:**
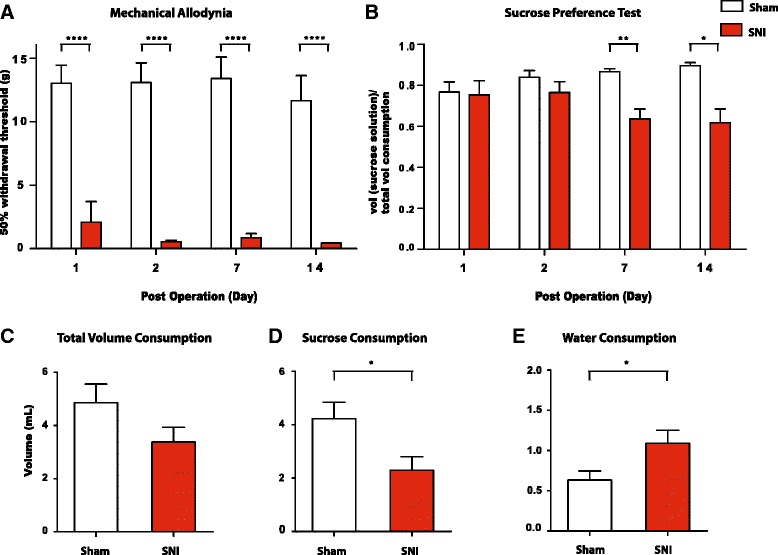
Spared nerve injury (SNI) causes persistent neuropathic pain and depression-like behaviors. **a** SNI-operated rats developed persistent mechanical allodynia after surgery, compared with sham-operated rats. Two-way ANOVA with Bonferroni post-test, *n* = 4–9, ****p < 0.0001. **b** SNI-treated rats developed worse sucrose preference than sham-treated rats on postoperative day 7 and 14. Two-way ANOVA with Bonferroni post-test, *n* = 4–10, *p < 0.05, **p < 0.01. **c** SNI caused no changes in total fluid consumption on the SPT 7 days after surgery. Student’s *t*-test, *n* = 9–10, *p* > 0.05. **d** SNI caused decreased sucrose consumption on the SPT 7 days after surgery. Student’s *t*-test, *n* = 9–10, *p < 0.05. **e** SNI caused increased water consumption on the SPT 7 days after surgery. Student’s *t*-test, *n* = 9–10, *p < 0.05. Error bars show mean and SEM

Recently, a number of studies have reported depression-like behaviors in rats with chronic neuropathic pain [16, 18]. Here, we used the SNI model to determine the time course of development of these depressive symptoms [16]. As previously reported, rats developed mechanical allodynia as early as 1 day after the SNI procedure, and this abnormal sensory hypersensitivity persisted for at least 14 days following SNI (Fig. 3a, *p* < 0.0001). In contrast, the control group that underwent sham operation did not show any sensory hypersensitivity (Fig. 3a).

Next, we measured sucrose preference after sham or SNI procedures. We found that both sham and SNI-treated rats showed a decreased preference for sucrose 1 day after surgery (Fig. 3b). In the case of sham-treated rats, incisional pain from sham surgery likely caused this short-term decrease in sucrose preference, similar to what we found with PI-treated rats (Fig. 1b). Sham-treated rats, however, quickly recovered their normal sucrose preference starting on postoperative day 2, likely because these rats began to recover from incisional pain. In contrast, sucrose preference decreased further in the SNI group over the next 7–14 days, as neuropathic pain from nerve injury worsened and became persistent or chronic (Fig. 3b). As the result, there were significant differences in sucrose preference on postoperative day 7 and 14 between these the sham and SNI groups (Fig. 3b, *p* < 0.05). These differences on SPT were due to decreased consumption of sucrose and increased consumption of water by the SNI group (Fig. 3d, e, *p* < 0.05). The total fluid consumption was not statistically different, however, in these two groups of rats (Fig. 3c).

### Post-incisional pain does not increase GluA1 levels at NAc synapses

**Fig. 4:**
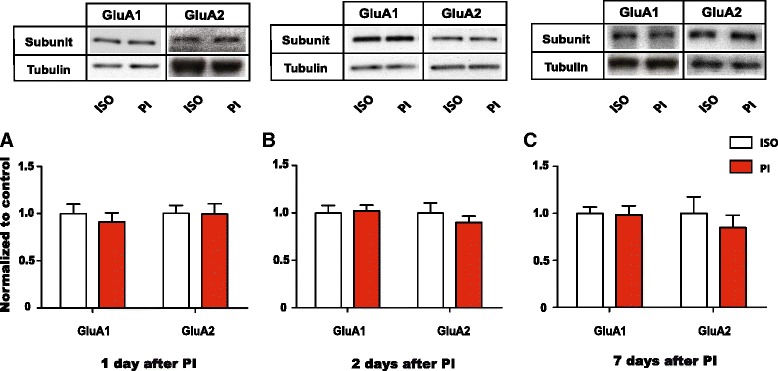
Paw incision does not cause an alteration in GluA1 and GluA2 levels at the synapses of the NAc. **a** Levels of GluA1 and GluA2 subunits in synaptoneurosome preparations from the NAc did not alter 1 day after PI. Two-way ANOVA with Bonferroni post-test, *n* = 6 (GluA1), *p* > 0.05; *n* = 6 (GluA2), *p* > 0.05. **b** Synaptic levels of GluA1 and GluA2 subunits did not alter 2 days after PI. Two-way ANOVA with Bonferroni post-test, *n* = 6 (GluA1), *p* > 0.05; *n* = 6 (GluA2), *p* > 0.05. **c** Synaptic levels of GluA1 and GluA2 subunits did not alter 7 days after PI. Two-way ANOVA with Bonferroni post-test, *n* = 6 (GluA1), *p* > 0.05; *n* = 6 (GluA2), *p* > 0.05. Data were normalized to values in the control group. Error bars show mean and SEM

Chronic neuropathic pain has been shown to selectively increase GluA1 levels in the NAc, and this increase contributes to the synaptic incorporation of GluA2-lacking receptors. Transmission through these GluA2-lacking receptors in turn reduces the depressive symptoms of pain [17]. Thus, GluA1 upregulation represents an important synaptic mechanism in the NAc to regulate the depressive symptoms of pain. Here we investigated whether this elevation in synaptic GluA1 levels is also a feature of reversible post-incisional pain. We used synaptoneurosome preparations to study AMPA receptor subunits at NAc synapses. Over 90 % of neurons in the NAc are medium spiny neurons, and synaptoneurosome preparations reflect synaptic fractions of these neurons. GluA1 and GluA2 are the most abundant AMPA receptor subunit types in the NAc. Thus, we measured the levels of GluA1 and GluA2 subunits from synaptoneurosome in the NAc using Western blots at different time points after PI. Interestingly, there were no statistically significant changes in the levels of GluA1 and GluA2 subunits between the PI and control groups 1 or 2 days after the onset of pain (Fig. 4a, b, *p* > 0.05). To make sure that GluA1 upregulation does not represent a delayed response after incision, we then measured GluA1 and GluA2 levels 7 days after PI. Again we found no alterations in GluA1/2 levels (Fig. 4c, *p* > 0.05). These results indicate that short-term post-incisional pain (of less than 7 days duration) does not trigger alterations in the AMPA receptor composition in the NAc.

### Inflammatory pain reversibly increases GluA1 levels at NAc synapses

**Fig. 5:**
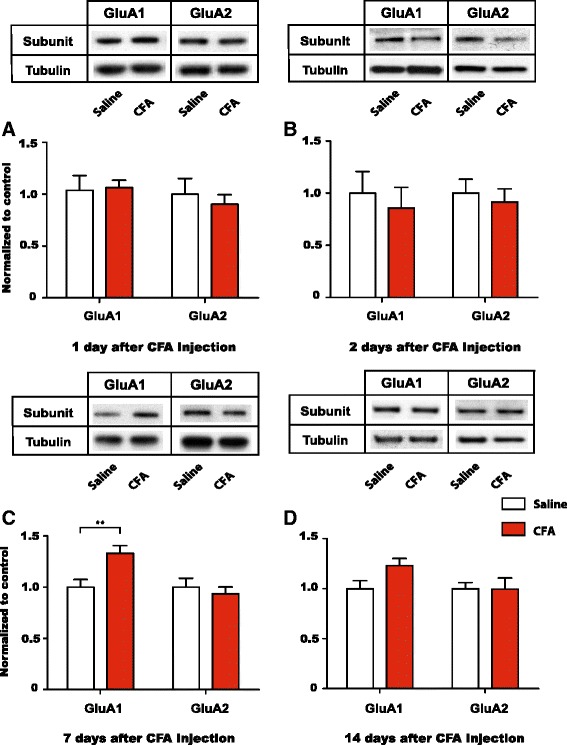
Persistent inflammatory pain causes a reversible increase in GluA1 levels at NAc synapses. **a** Levels of GluA1 and GluA2 subunits in synaptoneurosome preparations from the NAc did not alter 1 day after CFA injections. Two-way ANOVA with Bonferroni post-test, *n* = 5 (GluA1), *p* > 0.05; *n* = 6 (GluA2), *p* > 0.05. **b** Synaptic levels of GluA1 and GluA2 subunits in the NAc did not alter 2 days after CFA injections. Two-way ANOVA with Bonferroni post-test, *n* = 4–6 (GluA1), *p* > 0.05; *n* = 4–6 (GluA2), *p* > 0.05. **c** 7 days after CFA administration, levels of GluA1 were selectively increased at NAc synapses, whereas GluA2 levels were not altered. Two-way ANOVA with Bonferroni post-test, *n* = 8 (GluA1), **p < 0.01; *n* = 8 (GluA2), *p* > 0.05. **d** 14 days after CFA injection, GluA1 levels returned to control levels at NAc synapses, whereas GluA2 levels remained unchanged. Two-way ANOVA with Bonferroni post-test, *n* = 7–8 (GluA1), *p* > 0.05; *n* = 7–8 (GluA2), *p* > 0.05. Data were normalized to values in the control group. Error bars show mean and SEM

To understand if AMPA receptor plasticity in the NAc is unique to persistent neuropathic pain states, we evaluated the levels of GluA1 and GluA2 subunits at NAc synapses in the inflammatory pain (CFA) model. We found that shortly after the onset of pain (1 and 2 days after CFA injection), the levels of GluA1 and GluA2 were not altered (Fig. 5a, b, *p* > 0.05). This finding mirrors the finding from the PI model, in which post-incisional pain did not change GluA1/2 levels (Fig. 4a, b). However, 7 days after CFA injection, when pain had become persistent (Fig. 2), the levels of GluA1 subunits were increased (>30 %) in the CFA group compared with the control group (Fig. 5c, *p* < 0.01). GluA2 subunit levels, in comparison, remained unchanged. These findings indicate that changes in AMPA receptor levels are not restricted to neuropathic pain states. We then measured GluA1/2 levels 14 days after CFA/saline injections to see if this selective increase GluA1 levels at NAc synapses persisted even when pain had begun to subside in CFA-treated rats (Fig. 2). Interestingly, we found that at this time point, GluA1 levels also began to return to control levels (Fig. 5d, *p* > 0.05). These results suggest that GluA1 upregulation in the NAc occurs once pain has become persistent (>7 d), but that with the resolution of pain, GluA1 levels also return to normal.

**Fig. 6:**
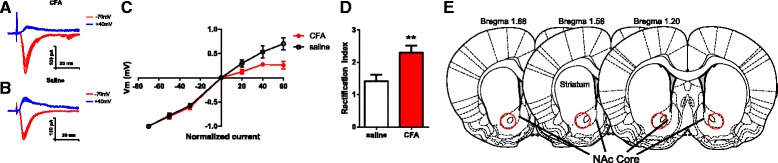
Persistent inflammatory pain causes the formation of GluA2-lacking AMPA receptors in the NAc. **a-c** Neurons in the NAc from rats that received CFA injection showed decreased EPSCs at depolarized voltages, compared with neurons from rats that received saline treatment. **a**, **b** Representative traces; **c** Normalized cumulative current–voltage relationship. **d** EPSCs from CFA-treated rats showed increased rectification index. Student’s *t* test, *n* = 10-12 neurons from 3 rats, **p < 0.01. **e** Schematic of the NAc showing EP recording sites in red circles. Recordings were done 7 days after either CFA or saline injection. Error bars show mean and SEM

A selective increase in synaptic level of GluA1 subunits, without a change in GluA2, indicates an increase in the overall number of AMPA receptors at synapses. Furthermore, this selective increase in GluA1 levels suggests the new formation of GluA2-lacking AMPA receptors, which are known to augment synaptic transmission due to higher conductance and the ability to trigger intracellular calcium signaling [40–42]. To confirm the formation of GluA2-lacking AMPA receptors in the persistent inflammatory pain state, we performed whole-cell patch clamp recordings from neurons in brain slices of the NAc 7 days after CFA or saline injection (Fig. 6). Excitatory postsynaptic currents (EPSCs) conducted by GluA2-lacking AMPA receptors display characteristic inward rectification due to channel block at depolarized potentials by endogenous polyamines [41]. We identified MSNs visually in the NAc core region, and recorded evoked EPSCs from these neurons (Fig. 6). Indeed, EPSCs from neurons in CFA-treated animals displayed an inward rectification, as indicated by nonlinearity in the current–voltage (I/V) relationship (Fig. 6a, c). This inward rectification was minimally expressed, however, in neurons from saline-treated rats (Fig. 6b, c). We then calculated the rectification index (RI) using I/V relationships for saline and CFA-treated animals [45]. When we compared the RIs for these two groups, we found a substantial difference (>50 % increase in the CFA group; *p* < 0.01, Fig. 6d). These electrophysiological data confirm the findings from our biochemical analysis and further indicate that persistent inflammatory pain leads to the formation of GluA2-lacking AMPA receptor currents in the NAc.

### Neuropathic pain causes persistent increases in GluA1 levels at NAc synapses

**Fig. 7:**
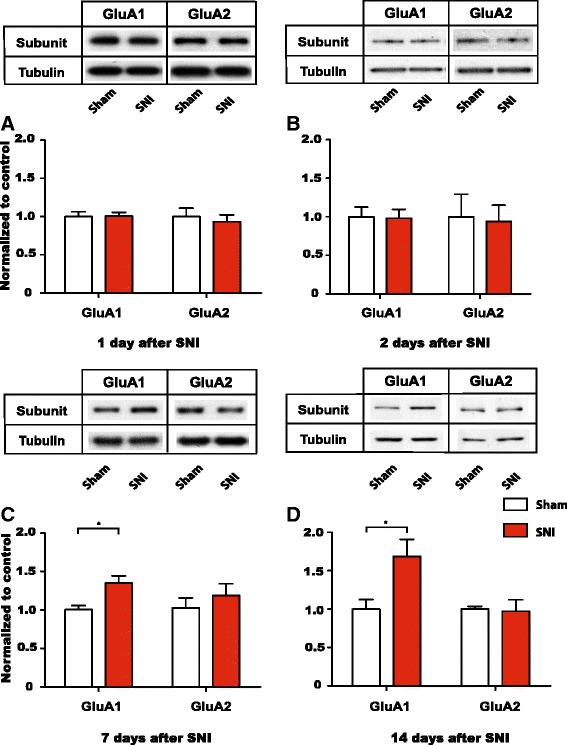
Chronic neuropathic pain causes a continued increase in GluA1 levels at NAc synapses. **a** Levels of GluA1 and GluA2 subunits in synaptoneurosome preparations from the NAc did not alter 1 day after SNI. Two-way ANOVA with Bonferroni post-test, *n* = 6 (GluA1), *p* > 0.05; *n* = 6 (GluA2), *p* > 0.05. **b** Synaptic levels of GluA1 and GluA2 subunits in the NAc did not alter 2 days after SNI. Two-way ANOVA with Bonferroni post-test, *n* = 6 (GluA1), *p* > 0.05; *n* = 6 (GluA2), *p* > 0.05. **c** 7 days after SNI, levels of GluA1 were selectively increased at NAc synapses, whereas GluA2 levels were not altered. Two-way ANOVA with Bonferroni post-test, *n* = 8–9 (GluA1), *p < 0.05; *n* = 5–6 (GluA2), *p* > 0.05. **d** 14 days after SNI, GluA1 levels continued to be elevated at NAc synapses, whereas GluA2 levels were unchanged. Two-way ANOVA with Bonferroni post-test, *n* = 5–7 (GluA1), *p < 0.05; *n* = 6 (GluA2), *p* > 0.05. Data were normalized to values in the control group. Error bars show mean and SEM

Chronic neuropathic pain has been shown to increase GluA1 expression and cause the formation of GluA2-lacking AMPA receptors at NAc synapses [17]. The time course of this GluA1 increase, however, has not been established. Our data on inflammatory pain suggests that GluA1 upregulation is a response to persistent pain. Thus, we hypothesized that GluA1 levels at NAc synapses would remain elevated as long as pain persisted. To test this hypothesis, we turned to the SNI model, which is known to cause pain lasting up to six months [16, 46]. We measured the levels of GluA1 and GluA2 subunits from synaptoneurosome preparations of NAc neurons 1, 2, 7 and 14 days after SNI or sham surgery. Concordant with our findings in PI and CFA models, we found no changes in the levels of GluA1 and GluA2 subunits between SNI and sham rats 1 or 2 days after surgery (Fig. 7a, b, *p* > 0.05). Compatible with what we found in the CFA model, however, GluA1 levels began to increase 7 days after SNI (Fig. 7c, *p* < 0.05). Furthermore, the level of increase (30 % from control levels) was quantitatively similar in CFA and SNI models at this time point. Meanwhile, as pain persisted 14 days after SNI (Fig. 3), GluA1 subunit levels continued to increase (>80 %, Fig. 7d, *p* < 0.05). GluA2 levels, in contrast, remained unaltered (Fig. 7a-d). These data support our hypothesis that GluA1 upreguation at NAc synapses represents an adaptive response to persistent pain. It further indicates that this form of synaptic plasticity occurs regardless of the peripheral source of pain.

### Persistent pain increases the trafficking of GluA1 to NAc synapses

**Fig. 8:**
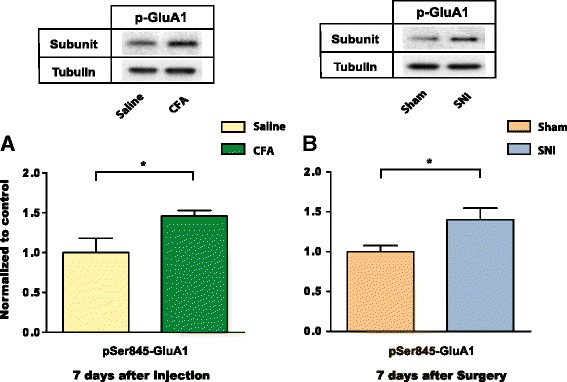
Persistent inflammatory pain and neuropathic pain increase pSer845-GluA1 levels at NAc synapse. **a** 7 days post injection, levels of pSer845-GluA1 increased in synaptoneurosome preparations from the NAc in CFA-treated rats. Student’s *t*-test, *n* = 6 (Saline); *n* = 6 (CFA), *p < 0.05. **b** 7 days post-surgery, levels of pSer845-GluA1 increased at NAc synapses in SNI-treated rats. Student’s *t*-test, *n* = 7 (Sham); *n* = 9 (SNI), *p < 0.05. Data were normalized to values in the control group. Error bars show mean and SEM

A common mechanism for increased AMPA receptor subunit expression at the synapse is increased exocytotic trafficking [47, 48]. To assess the role of trafficking, we examined the phosphorylation of GluA1 Ser845, which has previously been shown to be a critical step for the targeting of GluA1 to the synaptic surface [49]. Here, we found that the level of phospho-Ser845 in the synaptoneurosomes of NAc was increased by approximately the same amount as GluA1 subunit levels 7 days after the onset of pain in both CFA and SNI models (Fig. 8a, b, *p* < 0.05). These results suggest that this trafficking mechanism is conserved in the persistent pain state regardless of the peripheral cause of pain. Hence most likely phosphorylation at Ser845 has contributed to the trafficking of GluA1 subunits to the NAc synapses in persistent pain states.

### GluA2-lacking receptors in the NAc regulates depression-like behaviors associated with persistent pain

Most AMPA receptors contain GluA2 subunits. A selective increase in the synaptic level of GluA1 subunits without a concurrent change in the GluA2 level, however, has been shown to lead to the formation of GluA1 homomers, which are GluA2-lacking AMPA receptors [17, 40–42]. GluA2-lacking receptors display unique biophysical properties including Ca^2+^ permeability and high single unit conductance, and these receptors have been shown to regulate a host of behaviors [40–42], including the relief of depressive symptoms of persistent neuropathic pain in the SNI model [17].

**Fig. 9:**
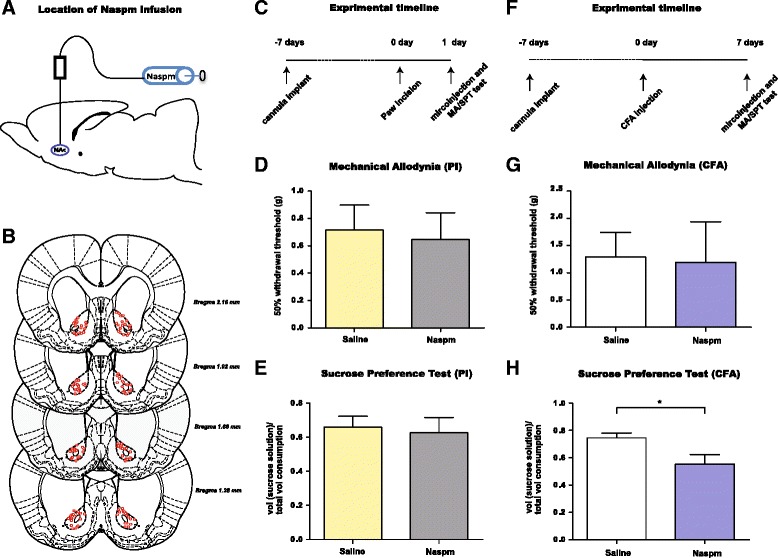
GluA2-lacking receptors in the NAc regulate depression-like behaviors induced by persistent inflammatory pain but not short-term postoperative pain. **a** Schematic showing Naspm infusion. **b** Placement of injectors depicted by red circles. **c** Timeline for experiments in PI-treated rats. **d**, Naspm infusion into the NAc core did not change mechanical hypersensitivity in PI-treated rats (1 day after procedure). Student’s *t*-test, *n* = 6 (Saline); *n* = 7 (Naspm), *p* > 0.05. **e** Naspm did not change sucrose preference in PI-treated rats. Student’s *t*-test, *n* = 8 (Saline); *n* = 8 (Naspm), *p* > 0.05. **f** Timeline for experiments in CFA-treated rats. **g** Naspm infusion into the NAc did not change mechanical hypersensitivity in CFA-treated rats (7 days after injection). Student’s *t*-test, *n* = 7 (Saline); *n* = 7 (Naspm), *p* > 0.05. **h** Naspm in the NAc reduced sucrose preference in CFA-treated rats. Student’s *t*-test, *n* = 9 (Saline); *n* = 8 (Naspm), *p < 0.05. Error bars show mean and SEM

In order to understand the roles of GluA1 subunits in PI and CFA models, we injected 1-naphthyl acetyl spermine/*N*-acetyl-spermine (Naspm), a highly selective blocker of GluA2-lacking AMPA receptors, into the NAc to examine the effect of blocking these receptors on sensory and depressive features of pain (Fig. 9a, b). In the PI model, our biochemical assays showed no increases in GluA1 levels, suggesting that no new GluA2-lacking receptors were formed shortly after surgical incision. Thus, it is not surprising that the infusion of Naspm, which selectively blocks GluA2-lacking receptors but not GluA2-containing receptors, did not have a significant effect on pain behaviors in this model (Fig. 9c-e).

Next, we examined the effect of Naspm on behavioral responses to persistent inflammatory pain. Our biochemical data suggest that 7 days after the onset of pain, GluA1 levels are selectively increased, allowing the formation of GluA2-lacking receptors at the NAc synapse (Figs. 5c, 6). We found that Naspm infusion into the NAc did not alter mechanical hypersensitivity 7 days after CFA injection (Fig. 9g, *p* > 0.05), suggesting that increased GluA1 subunits do not play a dominant role in regulating the sensory transmission of the pain signal. However, glutamate signaling is known to play an important role in depression [50–52], and increased transmission through AMPA receptors have been shown to confer antidepressant effects [16, 53–55]. Therefore, we next examined the effect of blocking GluA2-lacking AMPA receptors in the NAc on depression-like behaviors induced by persistent inflammatory pain using the SPT. Sucrose preference was decreased in CFA-treated rats compared with saline-treated rats (Fig. 2b). When we measured sucrose preference in the presence of Naspm treatment (vs. saline) in CFA-treated rats, however, we found that it was substantially further reduced (Fig. 9h, *p* < 0.05). Thus, blocking GluA2-lacking receptors in the NAc worsened anhedonia, a key feature of depression-like behaviors in CFA-treated rats. These results in the inflammatory pain model have two important implications. First, they confirm our biochemical and electrophysiological results (Figs. 5c and 6) and indicate that GluA2-lacking AMPA receptors are formed in response to persistent pain. Second, these results are similar to previously reported results in the SNI model [17], and together, these findings suggest that, *in vivo*, GluA1 subunits are trafficked to the NAc synapse to provide protection against depressive symptoms, possibly as an adaptive mechanism to persistent pain, regardless of the peripheral etiology of pain.

## Discussion

Pain is well-known to cause depressed mood [5–7]. The causal relationship between pain and depression, however, has not been completely established. In this study, we have found that in three different animal pain models, pain can cause depression-like behaviors in a time course that is compatible with the development of sensory allodynia. More importantly, we have found that GluA1 subunits of the AMPA receptors are selectively increased at the synapses of NAc only after pain has become persistent, and that increased GluA1 levels function to relieve depressive symptoms of pain.

Previous studies have shown that chronic neuropathic pain and inflammatory pain both cause depression-like behaviors [16–20], but this is the first report that post-incisional pain, which presents a comparably shorter duration of pain, also causes a similar depressive phenotype. Interestingly, the time course of the development of depressive symptoms closely mirrors that of sensory allodynia in the PI model. In this model, anhedonic symptoms developed and resolved at the same time point when allodynic symptoms developed and resolved.

In the persistent inflammatory pain (CFA) model, anhedonia also developed concurrently with allodynia. In this pain model, anhedonic symptoms resolved after 7 days, when allodynic symptoms also began to resolve. Qualitatively, these results indicate that both sensory and depressive symptoms of pain resolve after a period of time. Quantitatively, however, these data suggest that the affective symptoms of pain resolve more completely than the sensory symptoms of pain early in the process of recovery from peripheral inflammatory insult. Such findings are in fact compatible with what has been reported in the clinical literature on acute and chronic pain, where affective indices for pain have been suggested to normalize earlier than reports of sensory pain scores [1, 6, 56, 57].

In the chronic neuropathic pain (SNI) model, due to the initial incisional pain caused by sham surgery, the sham group also developed reversible anhedonic symptoms. Therefore, the difference in sucrose preference between the SNI and sham groups was not pronounced for the first two days. After two days, however, sham-treated rats recovered their normal hedonic response, just like PI-treated rats. SNI-treated rats, in contrast, continued to display anhedonic symptoms.

Overall, results from these three distinct rat models suggest that depressive symptoms consistently accompany sensory symptoms of pain. These results indicate that depression is caused by pain, and that depressive symptoms are an integral component of the pain experience. There is a growing interest in understanding the mechanisms regulating the affective component of pain, including depressed mood [19, 58, 59]. Our results here validate the use of rat models for the study of depressive pain symptoms.

An alternative explanation for our data is that depression and pain developed independently. However, this is unlikely in our study. In the current study, the time course of depression-like behaviors as shown by anhedonia on the SPT closely mirrors the time course of sensory allodynia in all three pain models. As soon as allodynia begins to diminish, signaling the resolution of post-incisional or inflammatory pain, the hedonic response of rats also returns to normal. Thus, the most likely explanation of our data is that pain directly causes depression-like behaviors. This explanation is also compatible with clinical observations [5, 7].

Prior imaging studies have identified the NAc as a brain region that undergoes changes in morphology and connectivity in response to both acute and chronic pain [21–23, 60]. The changes in the NAc that occur at the molecular and synaptic levels, however, are less well characterized in the context of pain. Chronic neuropathic pain has been shown to selectively increase GluA1 subunit levels at the NAc synapses, leading to the formation of GluA2-lacking AMPA receptors [17]. An important contribution in the current study is to define the exact time course of this crucial synaptic event. In the broader context, our work on glutamate signaling complements previous studies demonstrating the roles, within the NAc, of opioid signaling and dopamine signaling in regulating descending inhibition, stress-induced hyperalgesia, and negative reinforcement from pain-relief [61–64].

The synaptic incorporation of GluA1 subunits requires a series of highly regulated signaling steps involving sequential phosphorylation at a number of key residues [49, 65–68]. Thus, the synaptic incorporation of these receptors tends to be found under unique behavioral conditions such as repeated consumption of cocaine or sucrose, prolonged cocaine withdrawal, fear conditioning, and stress [32, 39, 45, 69–72]. Chronic neuropathic pain has been shown to selectively increase GluA1 subunit levels at the NAc synapses, leading to the formation of GluA2-lacking AMPA receptors [17]. Our results here suggest that this form of synaptic plasticity is not seen in acute pain and is thus specific to persistent or chronic pain states. Such synaptic plasticity, however, is not unique to neuropathic pain, as persistent inflammatory pain can also cause GluA1 upregulation. Furthermore, our data suggests that phosphorylation of Ser845 may be an important mechanism in the trafficking of GluA1 to the NAc synapse in the inflammatory pain model, similar to previous findings demonstrating the involvement of Ser845 phosphorylation in the synaptic targeting of GluA1 in the SNI model [17]. Thus, phosphorylation of Ser845 plays an important role in delivering GluA1 to the NAc synapses in the presence of persistent pain.

Our results in CFA and SNI models suggest that at least 7 days of persistent pain are required to increase GluA1 AMPA receptor subunits in the NAc. Interestingly, this time frame is almost identical to the amount of time required to increase GluA1 subunits in the NAc in the context of repeated consumption of a natural reward [70]. Natural rewards and pain represent opposite valence in the reward-aversion valuation spectrum. Indeed, GluA1 upregulation has been reported in a number of studies on rewards [45, 70, 73]. Interpreted in this context, our results suggest that rewards and pain provide similar stimuli to trigger AMPA receptor plasticity in the NAc. There is increasing evidence that the NAc codes both the salience and the valence of a stimulus [74–77]. Thus, it is not surprising that similar synaptic changes occur in response to both pain and rewards.

The comparison between the CFA and SNI models reveals that GluA1 upregulation is not permanent. Pain in the CFA model begins to resolve after 14 days. GluA1 levels at the synapse also return to normal at the same time. The level of GluA1 subunits at the NAc synapse remains elevated, however, in the SNI model, because pain also remains persistent 14 days after SNI. This comparison suggests that AMPA receptor signaling not only codes the intensity of an aversive stimulus, but it also codes the duration of this stimulus.

A selective increase in GluA1 subunits without alterations in GluA2 levels leads to the synaptic incorporation of GluA2-lacking AMPA receptors [40–42]. Delayed GluA1 upregulation has been described in the NAc with repeated consumption of natural rewards or drugs of addiction [70, 78], similar to AMPA receptor changes identified with prolonged disturbances of sensory systems [79]. In these models, homeostatic plasticity has been posited as a potential mechanism for the formation of GluA2-lacking receptors to regulate behavior. The length of time required to increase GluA1 levels in persistent pain states found in our study is compatible with the time course found in these other behavioral models. This similarity raises the intriguing possibility that the synaptic modification observed in persistent pain states may also represent a form of homeostatic plasticity in the reward system.

Our pharmacological experiments suggest that increased GluA1 levels in the NAc constrain the depressive symptoms of persistent inflammatory pain. Previous studies have identified a similar role for GluA1 in neuropathic pain states [17]. Thus, AMPA receptors play a key role in the regulation of depressive symptoms of pain regardless of the etiology of pain. In a broader sense, these results suggest that glutamate signaling in the NAc may be an important mechanism that links persistent pain with depression. The NAc receives glutamatergic inputs from the PFC, hippocampus, amygdala, and the thalamus [80]. Glutamate signaling in the PFC, amygdala and hippocampus has been studied in the regulation of depression [26–28, 35–37]. Interestingly, altered synaptic activities within these regions have also been demonstrated to regulate cognitive and affective responses to pain [81–85]. Thus, a glutamatergic circuit that involves the PFC, amygdala, hippocampus and NAc may form an important basis for understanding the connection between pain and depression. Future studies to further define the role of AMPA receptor signaling within these interconnected regions will likely result in the precise mapping of an affective pain circuit.

## Conclusion

Our study shows that in rats, post-incisional, inflammatory as well as neuropathic pain all can cause depression-like behaviors. However, GluA1 upregulation at the synapses of NAc signals an adaptive response only to persistent pain. Thus, AMPA receptor trafficking dynamics in the brain’s reward system plays an important role in the regulation of chronic pain, and GluA1 levels in the NAc can additionally serve as a molecular marker for the chronicity of pain.

## Materials and methods

### Animals

All procedures in this study were approved by the New York University School of Medicine Institutional Animal Care and Use Committee (IACUC) as consistent with the National Institute of Health (NIH) *Guide for the Care and Use of Laboratory Animals* (publication number 85–23) to ensure minimal animal use and discomfort. Male Sprague–Dawley rats were purchased from Taconic Farms, Albany, NY and kept at Mispro Biotech Services Facility in Alexandria Center for Life Science, with controlled humidity, room temperature, and 12-h (6:00 AM to 6:00 PM) light–dark cycle. Food and water were available *ad libitum*. Animals arrived to the animal facility at 250 to 300 g and were given on average 7 days to adjust to the new environment prior to the onset of any experiments.

### Animal surgeries and procedures

#### Paw Incisional (PI) surgery

The paw incisional surgery was performed as previously described [43], with a few minor modifications. Briefly, rats were anaesthetized with Isoflurane anesthesia (1.5–2 %), and the plantar surface of the right hind paw was sterilized and prepared. A 1.5 cm longitudinal incision was cut with a number 10 scalpel, through skin and fascia of the right plantar aspect of the paw. The incision started 0.5 cm from the proximal end of the heel and extended to the middle of the paw. The plantaris muscle was elevated and incised longitudinally. Gentle pressure was applied in order to cease bleeding and the wound was opposed with three single sutures using 5–0 nylon. The animals were allowed to recover in their home cages. Control rats only received Isoflurane anesthesia.

#### Complete Freund’s Adjuvant (CFA) administration

To produce inflammatory pain, CFA (mycobacterium tuberculosis, Sigma-Aldrich [St. Louis, MO], 0.1 ml) was suspended in an oil-saline (1:1) emulsion and injected subcutaneously into the plantar aspect of the hind paw. Control rats received an equal volume of saline injection.

#### Spared Nerve Injury (SNI) surgery

The spared nerve injury (SNI) surgery has been previously described in detail [46]. Briefly, under Isoflurane anesthesia (1.5–2 %), the skin on the lateral surface of the right thigh of the rat was incised and the biceps femoris muscle was dissected in order to expose three branches of the sciatic nerve: sural, common peroneal, and tibial. The common peroneal and tibial nerves were tied with non-absorbent 5.0 silk sutures at the point of trifurcation. The nerves were then cut distal to the knot, and about 3 to 5 mm of the distal ends were removed. In sham surgeries (control), the nerves mentioned above were dissected but not cut. Muscle and skin layers were then sutured closed in distinct layers.

#### Cannula implantation and intracranial injections

For cannula implantation, as described previously [86], rats were anesthetized with isofluorane (1.5–2 %). Rats were stereotaxically implanted with two 26-gauge guide cannulas (PlasticsOne, Roanoke, VA) bilaterally in the NAc core with coordinates: 1.6 mm anterior to bregma; 2.9 mm lateral to the sagittal suture, tips angled 8° toward the midline, 5.6 mm ventral to skull surface. Cannulas were held in place by dental acrylic and patency was maintained with occlusion stylets. For intracranial injections, solutions were loaded into two 30 cm lengths of PE-50 tubing attached at one end to 10-μl Hamilton syringes filled with distilled water and at the other end to 33-gauge injector cannula, which extended 2.0 mm beyond the implanted guides. Injection of solution then delivered bilaterally 0.5 ul of injection volume over a period of 100 s. Injector cannulas were kept in place for another 60 s prior to removal from guides to allow diffusion of solution into the brain. Following the removal of injector cannulas from cannula guides, stylets were replaced, and animals were subject for behavior tests. Behavior tests were done 15 min after intracranial injections. Following animal sacrifice, cryogenic brain sections were collected with thickness of 20 um using Microm HM525 Cryostat and analyzed for cannula localization with histological staining; animals with improper cannula placements were excluded from the study.

### Drugs

1-naphthyl acetyl spermine/*N*-acetyl-spermine (Naspm) was purchased from Sigma. Naspm was resuspended in saline to a concentration of 80 ug/ul. We injected 0.5 ul in each side. Intracranial injections were given at least 7 days after cannula implantation and were followed by behavioral tests. Equal volume of saline was injected as control.

### Animal Behavioral Tests

#### Mechanical hypersensitivity test

A traditional Dixon up-down method with von Frey filaments was used to measure mechanical hypersensitivity as described previously [16, 87, 88]. In brief, rats were individually placed into plexiglass chambers over a mesh table and acclimated for 20 min before the onset of examination. Beginning with 2.55 g, von Frey filaments in a set with logarithmically incremental stiffness (0.45, 0.75, 1.20, 2.55, 4.40, 6.10, 10.50, 15.10 g) were applied vertically to the plantar surface of the right paw, adjacent to the wound of rats after PI. For SNI and sham group, von Frey filaments were applied to the lateral 1/3 of right paws (in the distribution of the sural nerve) of rats after SNI or sham surgery. Similar tests were done on CFA- or saline-treated rats. 50 % withdrawal threshold was calculated as described previously [16].

#### Sucrose preference test

As described previously [16], animals were acclimated to the test room for at least 20 min. Two bottles (1 % sucrose solution vs. water) were presented to each animal for 1 h. At the end of each test, sucrose preference was calculated as volume of sucrose consumed divided by total liquid consumption for each rat. Rats were allowed to eat *ad libitum* prior the test.

### Subcellular fractionation and Western blotting

Rats were anesthetized with Isoflurane (1.5-2 %) and decapitated immediately. Brains were quickly removed and NAcs were collected on ice. The NAcs were dissected from 1.08 to 2.52 mm anterior to Bregma, with average sample weight of 40 mg. Synaptosome fractions were prepared as described previously [89, 90]. To prepare synaptoneurosome fractions, nucleus accumbens samples were homogenized in an ice-cold solution A (0.32 M sucrose, 1 mM NaHCO_3_, 1 mM MgCl_2_, 0.5 mM CaCl_2_, 0.1 mM PMSF and 1x Complete Protease Inhibitors; Roche Applied Science). Homogenates were centrifuged at 4,000 rpm for 10 min. The supernatant was collected and the pellet rehomogenized in solution A and centrifuged again at 3,000 rpm for 10 min. Combined supernatants were subjected to a second centrifugation at 3,000 rpm for 10 min. Supernatants were then spun at 14,000 rpm for 30 min. Pellet was resuspended in solution B (0.32 M sucrose, 1 mM NaHCO_3_) and homogenized. Homogenate was layered on top of a 5 mL 1 M sucrose and 1.2 M sucrose gradient and centrifuged at 30,000 rpm for 2 h. Purified synaptosomes were collected at the 1 M and 1.2 M sucrose interface, suspended in solution B and centrifuged at 40,000 rpm for 45 min. Synaptosomal pellets were resuspended in 25 mM TRIS with 4 % SDS. Equal amounts of fractions were loaded on SDS-PAGE gels and analyzed by Western blot as previously described previously [89, 90]. The following antibodies were used: GluA1 (1:1000, Millipore), phospho-Ser845GluA1 (1:1000, Millipore), GluA2 (1:1000, Millipore) and tubulin (1:30,000, Sigma-Aldrich).

### Electrophysiology

Rats were deeply anesthetized with isofluorane (2 %) and decapitated immediately. Brains were quickly removed into dissection buffer consisted of the following (in mM): 75 sucrose, 87 NaCl, 2.5 KCl, 1.25 NaH_2_PO_4_, 0.5 CaCl_2_, 7 MgCl_2_ 6 H_2_O, 25 NaHCO_3_, 10 dextrose, bubbled with 95 % O_2_/5 % CO_2_ (pH 7.4). Coronal slices (300 μm thick) containing the NAc were cut in ice-cold dissection buffer using a vibrotome (Leica, VT1200S). The slices were then transferred to an incubation chamber containing warm dissection buffer for <30mins, and then moved into a warm ACSF (ACSF, in mM: 124 NaCl, 2.5 KCl, 1.25 NaH_2_PO_4_, 2.5 CaCl_2_, 1.5 MgSO_4_ 7H_2_O, 26 NaHCO_3_, and 10 dextrose) solution and allowed to return to room temperature for at least 1 h to allow for recovery. Slices were transferred to the recording chamber and perfused (2.0–2.5 ml min − 1) with oxygenated ACSF at 33-35 °C containing 25 μm APV and 10 μm picrotoxin to isolate EPSCs. Somatic whole-cell recordings were made from NAc core region medium spiny neurons in voltage-clamp with a Multiclamp 700B amplifier (Molecular Devices) using IR-DIC video microscopy. Patch pipettes (4–6 MΩ) were filled with intracellular solution (in mM: 125 Cs-gluconate, 2 CsCl, 5 TEA-Cl, 4 Mg-ATP, 0.3 GTP, 10 phosphocreatine, 10 HEPES, 0.5 EGTA, 100 μM spermine, and 3.5 QX-314). Data were filtered at 2 kHz, digitized at 10 kHz, and analyzed with Clampfit 10 (Molecular Devices). Extracellular stimulation (0.01-1 ms, 5–150 μA) was applied with a small glass bipolar electrode 0.05-0.5 mm from the recording electrode. EPSC amplitude was measured at holding potentials of −70, −50, −30, 0, +20, +40 and +60 mV. The rectification index (i_r_) was calculated by correcting any potential shifts in reversal potential and computed from the following equation: i_r_ = (I_−70_/ 70)/(I_+40_ / 40), where I-70 and I + 40 are the EPSC amplitudes recorded at −70 mV and +40 mV, respectively [45].

### Data analysis and statistics

Two-way ANOVA with repeated measures, followed by *post hoc* multiple pair-wise comparison Bonferroni test was used to compare the 50 % withdrawal threshold of PI vs. control rats; CFA vs. saline-treated rats and SNI vs. sham rats. SPT was also analyzed using the two-way ANOVA test with repeated measures and post hoc multiple pair-wise comparison Bonferroni test. For total fluid consumption, sucrose consumption and water consumption on the SPT, an unpaired two-tailed Student’s *t*-test was used to analyze performances of PI vs. control group; CFA vs. saline-treated group and SNI vs. sham group. For Western blots, two-way ANOVA with *post hoc* multiple pair-wise comparison Bonferroni test was also used to compare the proteins levels of GluA1 and GluA2 in PI vs. control animals; CFA vs. saline-treated animals and SNI vs. sham animals. An unpaired two-tailed Student’s *t*-test was used to analyze the level of phospho-Ser845GluA1 in CFA vs. saline and SNI vs. sham rats. For electrophysiology experiments, difference in rectification index between CFA and control group was analyzed using unpaired two-tailed Student’s *t*-test. An unpaired two-tailed Student’s *t*-test was used to compare results on the SPT between Naspm and saline treatment in PI- and CFA-treated rats. For all tests, a p value <0.05 was considered statistically significant. All data were analyzed using GraphPad Prism Version 6 software (GraphPad, La Jolla, CA).
